# The Changing Landscape of Anticoagulation in Pediatric Extracorporeal Membrane Oxygenation: Use of the Direct Thrombin Inhibitors

**DOI:** 10.3389/fmed.2022.887199

**Published:** 2022-07-06

**Authors:** Cindy Neunert, Meera Chitlur, Cornelia Heleen van Ommen

**Affiliations:** ^1^Department of Pediatrics, Columbia University Medical Center, New York, NY, United States; ^2^Division of Hematology, Oncology, Carmen and Ann Adams Department of Pediatrics, Children’s Hospital of Michigan, Central Michigan University, Detroit, MI, United States; ^3^Department of Pediatric Hematology and Oncology, Erasmus Medical Center University Medical Center Sophia Children’s Hospital, Rotterdam, Netherlands

**Keywords:** extracorporeal membrane oxygenation, anticoagulation, unfractionated heparin, bivalirudin, pediatric, argatroban

## Abstract

Bleeding and thrombosis frequently occur in pediatric patients with extracorporeal membrane oxygenation (ECMO) therapy. Until now, most patients are anticoagulated with unfractionated heparin (UFH). However, heparin has many disadvantages, such as binding to other plasma proteins and endothelial cells in addition to antithrombin, causing an unpredictable response, challenging monitoring, development of heparin resistance, and risk of heparin-induced thrombocytopenia (HIT). Direct thrombin inhibitors (DTIs), such as bivalirudin and argatroban, might be a good alternative. This review will discuss the use of both UFH and DTIs in pediatric patients with ECMO therapy.

## Introduction

Extracorporeal membrane oxygenation (ECMO) is increasingly used in pediatric patients with life-threatening cardiac and/or respiratory failure. Very recently, the extracorporeal life support organization (ELSO) reported 154,106 ECMO runs by 521 participating centers worldwide since 1990 (1). Neonatal and pediatric ECMO runs accounted for 29.4 and 20.1% of the total number of ECMO runs, respectively. ECMO is generally indicated in patients with acute severe heart or lung failure with high mortality risk despite optimal conventional therapy. Indications for pediatric ECMO include a reversible disease process in which ECMO provides a short-term bridge to recovery. In some cases, ECMO can be used as a bridge to transplantation. In the study of Dalton et al. bleeding complications, such as intracranial hemorrhage, were seen in up to 70.2% of neonatal and pediatric patients with ECMO. Thrombotic complications, such as circuit thrombosis and cerebral infarction, occurred in up to 37.5% of neonatal and pediatric patients. Despite increasing clinical expertise and improvements in technology, hemostatic complications, such as bleeding and thrombosis, remain an important cause of mortality and morbidity in ECMO-treated children worldwide. The hemostatic complications are caused by both circuit and systemic patient factors, which influence the unique balance of the hemostatic system (3). They commence upon the exposure of blood to the foreign, non-endothelial materials of the extracorporeal circuit, initiating activation of coagulation, and acute inflammatory responses, shifting the hemostatic balance to a hypercoagulable state. Antithrombotic therapy is necessary to maintain the patency of the circuit and to reduce thrombotic complications while minimizing bleeding. Until 2018, most centers used unfractionated heparin (UFH). Since then, the use of direct thrombin inhibitors (DTI), especially bivalirudin and argatroban, has increased. In this review, we will discuss the use of both UFH and DTIs in pediatric patients who received ECMO therapy.

## Unfractionated Heparin

### Characteristics of Unfractionated Heparin

Until recently, all patients with ECMO were anticoagulated with UFH, mainly because of the long-term experience with the anticoagulant, the lack of better alternatives, and the ability to rapidly reverse with protamine sulfate when complications occur. UFH is a sulfated mucopolysaccharide. Heparin molecules range in molecular weight and have a mean molecular weight of 15,000 kDa, corresponding to about 45 saccharide units (4). About one-third of the heparin molecules possess the unique pentasaccharide sequence, responsible for its anticoagulant effect. Via this pentasaccharide sequence, UFH binds to antithrombin, causing a conformational change and increasing antithrombin efficiency by a 1,000-fold, to inhibit thrombin (factor IIa) and factors Xa, IXa, XIa, and XIIa. The heparin-antithrombin complex is, however, unable to inactivate thrombin bound to fibrin. By inactivating free thrombin, UFH prevents both fibrin formation and thrombin-induced activation of platelets and factors V, VIII, and XI. For inhibition of thrombin, heparin should bind to both thrombin and antithrombin. Therefore, heparin molecules with less than 18 saccharides are too short to bridge antithrombin to thrombin and only inhibit factor Xa. Heparin is administered parenterally by continuous intravenous infusion or subcutaneous injection. Unfortunately, UFH binds to endothelial cells and endogenous plasma proteins other than antithrombin, contributing to the variability of the anticoagulant response to heparin among patients. The half-life of UFH depends on the dose and varies between 30 and 150 min, as low doses of heparin are rapidly cleared from plasma through binding to endothelial cell receptors and macrophages, whereas high doses of heparin are mostly cleared through the slower mechanism of renal clearance (4).

### Dosing and Monitoring

International surveys have shown large variation in the management of anticoagulation during ECMO (5, 6). The ELSO anticoagulation guidelines of 2014 recommend an initial UFH bolus of 50–100 units per kilogram body weight at the time of cannulation followed by a continuous infusion during the ECMO course (7). Close monitoring is required due to the variable anticoagulant effect of UFH, hemodilution, and coagulopathy of the patient due to underlying diseases and post-surgical conditions. There is no consensus on heparin dosing and monitoring and as a consequence, significant inter-institutional variability exists (6). The most commonly used coagulation tests include the activated clotting time (ACT), the activated partial thromboplastin time (aPTT), and the anti-factor Xa assay. All coagulation tests have limitations. ACT does not only reflect the effect of heparin but is also prolonged as a result of thrombocytopenia, hemodilution, hypothermia, low fibrinogen, and other clotting factor deficiencies. Using ACT alone in pediatric ECMO patients with UFH has been shown to lead to suboptimal anticoagulation (8). The baseline aPTT is higher in neonates and infants than in teenagers (9). In addition, the aPTT response to UFH is age-dependent, younger children having higher aPTT for the same anti-factor Xa (10). Prolongation of aPTT is not only caused by heparin administration but may also occur due to underlying conditions, such as diffuse intravascular coagulation. Furthermore, many aPTT reagents are available, and all coagulation laboratories should calibrate their assays to develop the target aPTT range. A meta-analysis of pediatric studies showed a very weak correlation between ACT and heparin dose and aPTT and heparin dose, respectively (11). Anti-factor Xa assay was the only laboratory test that showed a strong correlation with heparin dosing (*r* = 0.61; 95% CI 0.25–0.82).

A recent literature review investigated the association between coagulation tests and hemostatic complications, such as bleeding and thrombotic events (12). In nine studies, no association was found between aPTT or ACT or thromboelastography (TEG) and hemostatic complications. In one study, however, higher anti-factor Xa levels were associated with fewer clotting events (13). Furthermore, Northrop et al. showed that after incorporation of anti-factor Xa assay, TEG and antithrombin measurements in addition to the standard laboratory tests ACT, and aPTT in their revised anticoagulation protocol, the median blood product usage, and the frequency of cannula and surgical site bleedings decreased (14). In addition, the median circuit life was increased significantly from 3.6 to 4.3 days. Niebler et al. also showed a significant decrease in circuit changes and intracranial bleeds after changing from an ACT-based anticoagulation protocol to an anti-factor Xa-based protocol (15). Based on the abovementioned data, the anti-factor Xa assay seems to be the most useful test to monitor anticoagulation in patients with ECMO.

### Limitations Heparin

Although UFH has been used for years in patients with ECMO, it has various important limitations, especially in neonates and young infants (4). As mentioned before, UFH binds not only to antithrombin but also to other plasma proteins and endothelial cells. As the plasma concentrations of these proteins depend on age and underlying conditions of the patient, the heparin response is unpredictable and needs to be closely monitored. Monitoring of UFH is a challenge, as previously explained. Another limitation of UFH is the development of heparin resistance, which is defined by a progressive increase of heparin dose based on anti-factor Xa, aPTT, or ACT levels. Several mechanisms may be responsible for this phenomenon, i.e., decreased levels of antithrombin, increased binding to proteins or platelets, or increased factor VIII. Decreased levels of antithrombin may be seen in several settings that include neonates, nephrotic syndrome, and consumption and insufficient synthesis in critically ill patients; all of which can be present in the ECMO population. UFH may cause bone loss by decreasing bone formation. However, as UFH is usually given for a short period of time, its adverse effect on the bone will probably be negligible. Finally, heparin may bind to platelet factor 4 (PF4), leading to the formation of heparin-PF4 antibodies, which activate platelets, causing heparin-induced thrombocytopenia (HIT). In pediatric patients with ECMO, this is a rare condition. When HIT is suspected, UFH should be stopped immediately and alternative anticoagulation initiated to maintain the patency of the circuit and to treat HIT. With the development of DTIs, such as bivalirudin and argatroban, an alternative to UFH has become available in ECMO patients with HIT. These anticoagulants might also be promising in patients with ECMO in general. See Table 1 for features of UFH and bivalirudin.

**TABLE 1:** Features of unfractionated heparin and bivalirudin.

	Unfractionated heparin	Bivalirudin
Molecular weight	∼ 15,000 Heterogeneous mixture	2,180
Main target	Factor Xa, factor IIa	Direct thrombin inhibition
Activity against thrombin	Inhibits thrombin not bound to fibrin	Inhibits bound and unbound thrombin
Antithrombin dependency	Yes	No
Half-life	30–150 min (dose-dependent)	25 min
Route of administration	Intravenous	Intravenous
Protein binding	Non-specific binding to serine proteases	None
Metabolism/excretion	Reticuloendothelial system and excretion by kidneys	80% serum proteases 20% renal excretion
Use in renal failure	Yes	Dose reduction and careful monitoring
Monitoring	Anti-Xa assay aPTT	aPTT dTT
Reversibility	Protamine	Lack of reversal agent (short half-life)
Risk of HIT	Yes, but low absolute risk	No risk
Risk of osteoporosis	Yes, after long-term use	No
Costs	Inexpensive	Expensive

*aPTT, activated partial thromboplastin time; dTT, diluted thrombin time; HIT, heparin-induced thrombocytopenia.*

## Direct Thrombin Inhibitors: Bivalirudin

### Characteristics of Bivalirudin

Bivalirudin is a synthetic DTI that binds reversibly to thrombin *via* both the active/catalytic site and the exosite 1/fibrinogen binding site, independent of antithrombin. It is a small peptide, with a molecular weight of approx. 4,000 Da, which is cleaved by proteases that includes thrombin (16). It has a short half-life of 25 min and approximately 80% is enzymatically cleared and the rest is renally eliminated, allowing its use in patients with mild-to-moderate renal dysfunction without dose modification, but the half-life can be prolonged to 60 min in patients with renal failure requiring hemodialysis (16). Unlike UFH, bivalirudin is able to inhibit thrombin in circulation and clot-bound thrombin thereby decreasing clot stability and promoting thrombolysis. Unlike UFH, bivalirudin does not bind to other circulating plasma proteins and therefore its activity is more predictable. It is also not inhibited by platelet factor 4 (PF4) and potentially also inhibits platelet activation by inhibiting thrombin and in turn activation of factors V, VIII, and X (17). The lack of an antidote/reversal agent is a major disadvantage. While the short half-life is deemed an advantage, in situations associated with stasis, this may prove to be a disadvantage. Therefore, choosing the right anticoagulant for the right patient is crucial.

### Monitoring of Bivalirudin

Monitoring anticoagulation can be extremely challenging in extremely sick children where the risk for both bleeding and thrombosis is high. The DTIs act like a factor inhibitor in coagulation-based assays and therefore lead to an underestimation of factor activities and overestimation of protein C and protein S activities (18). By inhibiting thrombin, bivalirudin results in the prolongation of the PT, aPTT, thrombin time (TT), and ACT. The aPTT is often the most readily available assay and therefore is often used for monitoring of bivalirudin with the recommended target range being 1.5–2.5 times the baseline aPTT.

The aPTT assay has several disadvantages, as has been noted with heparin. It has been well established that at high bivalirudin concentrations, the aPTT does not show a linear correlation and there is a plateau effect and may therefore place the patient at risk for bleeding (18). In addition to this, it has been well established that the aPTT is unreliable in patients with lupus anticoagulants or with other factor deficiencies, and increased concentrations of coagulation proteins especially factor VIII, common in really sick patients like those on ECMO, result in significant variability of the aPTT (19). Traditionally, it has been noted that the PT does not correlate with bivalirudin dose, especially at higher doses, and therefore is not used to monitor bivalirudin; however, a recent single center prospective review of bivalirudin use in pediatric ECMO by Ryerson et al. reported that they saw a statistically significant correlation between the international normalized ratio (INR) and bivalirudin dose (20). This has not been reported by others and will require further studies. The TT, on the other hand, is noted to be too sensitive and therefore not a good measure of bivalirudin anticoagulation, but can be used to screen the patient prior to invasive procedures, to rule out the presence of even low concentrations of a DTI (21). The anti-factor IIa assay measures the amount of residual thrombin activity in a sample anticoagulated with bivalirudin, which will be inversely proportional to the amount of bivalirudin in the sample. This assay is not affected by the presence of lupus anticoagulants or factor deficiencies. It is currently not FDA approved for monitoring of DTIs and is therefore it is also not readily available in all laboratories. The therapeutic range is still to be established.

In addition to the routine assays, tests that measure the content of DTI in the plasma are another option. These assays include the ecarin clotting time (ECA) and diluted thrombin time (dTT). A recent study by Beyer et al. demonstrated a significant discordance between the aPTT and the ECA and dTT with a higher rate of bleeding complications in patients whose DTI dose was titrated exclusively based on the aPTT (22). It appears that there is a growing body of evidence to support the elimination of the use of the aPTT alone to monitor DTIs but there is a lack of supporting evidence to show poor outcomes. Hence, the aPTT continues to be used to monitor bivalirudin and other DTIs.

Ecarin is a metalloprotease isolated from viper venom, which directly activates thrombin and is therefore not affected by other factor deficiencies or lupus anticoagulants. The measured clotting time (CT) is theoretically directly proportional to the concentration of the DTI. However, studies showed that it was only suitable for bivalirudin but not lepirudin or argatroban due to the sensitivity of the chromogenic substrate chosen (23). The dTT assay is a modification of the TT. Since the routine TT is too sensitive to the presence of a DTI, diluting the plasma increases the sensitivity of the assay and allows a linear correlation between the concentration of the DTI and the dTT. Both the ECA and the dTT have been shown to have a more linear correlation to the DTI concentration and are independent of the prothrombin concentration in the plasma (22). Despite these advantages, the exact relationship between the drug concentration and the outcomes of bleeding and thrombosis remains to be established, especially since these assays are limited to very specialized labs.

Global coagulation assays TEG and thromboelastometry (ROTEM) are 2 whole blood coagulation assays that are currently being studied for their utility in monitoring the anticoagulation of patients on mechanical circulatory devices. They are able to measure the changes in viscosity of dynamics of clot formation. It remains an assay that is utilized in major centers and therefore has limited data. Studies have shown a good correlation between the anti-factor II assay and an ecarin-modified TEG (24). Similarly, another study found a correlation between the CT of the ROTEM with both intrinsic pathway activator (INTEM) and CT with hepzyme (HEPTEM) and the aPTT and Hepzyme aPTT (25). Data are still scarce and no guidance is available for therapeutic levels. It seems unfortunate, however, that the comparisons are still with the aPTT, an assay that has been shown to be inaccurate in these situations.

### Dosing of Bivalirudin

There are no guidelines for dosing of bivalirudin in ECMO. Dosing strategies vary significantly by institutions. In adults, the majority of the studies report doses varying from 0.025 to 0.05 mg/kg/h with the average rate of bivalirudin infusion required to maintain therapeutic aPTT or ACT levels varying from 0.028 to 0.5 mg/kg/h (26). There is also no consensus on whether a loading dose should be used or not. In studies comparing the 2 strategies, the difference in time to achieving therapeutic levels was only 4 h (27). Further studies are required to determine the safety and risk for bleeding with bolus dosing. In pediatric patients, the largest study by Hamzah et al. reported starting with an infusion rate of 0.3 mg/kg/h for those with creatinine clearance of > 60 ml/min or 0.15 mg/kg/h for those with renal dysfunction. Infusion rates of 0.05–0.3 mg/kg/h were reported to maintain therapeutic aPTT (28). These studies showed both the safety and feasibility to use bivalirudin for patients on ECMO.

It has also been shown that bivalirudin requirements increase with time. Hamzah et al. indicated that improved renal function with ECMO, upregulation of proteases that cleave thrombin resulting in increased thrombin levels, increased clot burden over time in the circuit, and increasing levels of fibrinogen over time resulting in increased competition for thrombin binding as possible reasons for this phenomenon. They also reported a dose-dependent increase in PT/INR, which may be suggestive of effects on other coagulation factors beyond thrombin.

### Label Indication

Bivalirudin is currently approved for patients who underwent percutaneous coronary intervention (PCI), i.e., patients with or at risk for having HIT or heparin-induced thrombocytopenia with thrombosis syndrome (HITTS). Initial US Food and Drug Administration (FDA) approval was based on results from the Hirulog Angioplasty Study (HAS) where 4,098 patients were randomized to receive bivalirudin or UFH during angioplasty for unstable angina or post-infarct angina (bivalirudin *n* = 2,059, UFH *n* = 2,039). Bivalirudin showed no benefit over UFH with regards to the primary composite outcome of any of the following hospital and procedural complications: death, myocardial infarction, the abrupt closure of the dilated vessel, or rapid clinical deterioration of cardiac origin requiring bypass surgery, intra-aortic balloon counter-pulsation, or repeated coronary angioplasty (11.4 vs. 12.2%; *p* = 0.44) (29). However, patients receiving bivalirudin demonstrated a lower incidence of major hemorrhage (3.8% vs. 9.8%; *p* < 0.001). Follow-up analysis that included an intention to treat the model with the 214 patients not included in the original analysis showed similar results with regard to ischemic and hemorrhagic complications, with some slight increase in benefit seen with bivalirudin based on an adjusted primary end point of death, myocardial infarction, and revascularization (6.2% vs. 7.9%; *p* = 0.039) (30). Thus, bivalirudin is at least equitable to UFH with regard to ischemic complications but has a potential benefit of providing lower levels of systemic anticoagulation resulting in a reduction in bleeding rates.

Several subsequent studies expanded the use of bivalirudin to PCI in the setting of glycoprotein IIB/IIIa antagonists. The pilot trial, Comparison of Abciximab Complications with Hirulog for Ischemic Events Trial (CACHET) established the proper dosing regimen for bivalirudin for PCI in this setting (0.75 mg/kg bolus; 1.75 mg/kg/h for the duration of the procedure) (31). This dose was applied in the Randomized Evaluation in PCI Linking Angiomax to Reduced Clinical Events trial (REPLACE-1) and the larger REPLACE-2 trial (32, 33). REPLACE-2 (*n* = 6,010) successfully met the non-inferiority end point as compared to heparin with regards to the composite outcome of death, myocardial infarction (MI), urgent revascularization, or in-hospital major bleeding within 30 days [9.2% bivalirudin vs. 10% controls, odds ratio (OR) 0.92; 95% CI 0.77–1.09; *p* = 0.03]. Bivalirudin was also found to have lower rates of major bleeding (2.4% vs. 4.1%; *p* < 0.001) and fewer patients treated with bivalirudin experienced a decline in platelet count below < 100 × 10/^9^l (0.7% vs. 1.7%; *p* < 0.001).

Bivalirudin for PCI in patients with HIT was investigated in the anticoagulation therapy with bivalirudin to assist in the performance of PCI in patients with heparin-induced thrombocytopenia trial (ATBAT) (34). Fifty-two patients with either a new diagnosis of HIT or a past history of HIT were treated with bivalirudin. Procedural success (TIMI grade 3 flow and < 50% stenosis) was achieved in 98% of patients, and clinical success (absence of death, emergency bypass surgery, or Q-wave infarction) in 96%.

### Off-Label Use

Bivalirudin has increasingly been used off-label in part because it has a relatively short half-life and predominantly non-organ-independent clearance with less need for reduction in the setting of mild or moderate renal function. Additionally, it is not dependent on a co-factor and therefore less likely to result in drug resistance as can be seen with heparin and low levels of antithrombin (35). For all these reasons, it has been favored for off-label use in cardiac patient management and the management of patients with HIT/HITTS. Off-label use and a few highlighted studies are shown in Table 2. These include medical management of acute coronary syndrome, cardiopulmonary bypass (CBP) on and off pump, and HIT/HITTS with or without the need for cardiac intervention.

**TABLE 2:** Bivalirudin trials in adults.

Indication	Study	Study design	Number	Bivalirudin	Comparator	Primary outcome	Results
Acute coronary syndrome medical management							
	ACUITY trial (52)		13,819	Bivalirudin with (Group B) or without GPIIa/IIIa therapy (Group C)	UFH or LMWH with GPIIa/IIIa therapy (Group A)	Net clinical outcome: death, MI, unplanned revascularization at 30 days, and major bleeding	Group B vs. Group A: non-inferior (11.8% vs. 11.7%) Group C vs. Group A: non-inferior (10.1% vs. 11.7%; *p* = 0.02; RR 0.86; 95% CI 0.77–0.97; *p* = 0.02)
Coronary artery bypass							
	EVOLUTION-OFF* (53) *Off pump coronary artery bypass	Randomized, open-label multicenter	157	Bivalirudin	UFH with protamine reversal	Procedural success: absence of death, Q-wave MI, repeat coronary revascularization and stroke at day 7 or discharge	No difference in the primary outcome (93%) Stroke higher in the UFH group (5.5% vs. 0%; *p* = 0.05) No difference in total blood loss and reoperations for bleeding
	EVOLUTION-ON (54)	Randomized, open-label multicenter	150	Bivalirudin	UFH with protamine reversal	Procedural success: absence of death, Q-wave MI, repeat coronary revascularization and stroke at day 7 or discharge	No difference in the outcome between bivalirudin and UFH at 7 days (94.9% vs. 96.2%), 30 days (94.9% vs. 94.2%), or 12 weeks (94.8% vs. 92.2%)
HIT/HITTS							
	CHOOSE-ON (55)	Prospective open-label multicenter	50	Bivalirudin	None	Procedural success: absence of death, Q-wave MI, repeat coronary revascularization and stroke at day 7 or discharge	94%
	CHOOSE-OFF (56)	Prospective open-label multicenter	35	Bivalirudin	None	Procedural success: absence of death, Q-wave MI, repeat coronary revascularization and stroke at day 7 or discharge	92% of patients

*UFH, unfractionated heparin; LMWH, low molecular weight heparin; MI, myocardial infarction; HIT, heparin-induced thrombocytopenia; HITTS, heparin-induced thrombotic thrombocytopenia syndrome.*

### Pediatric Use

Bivalirudin is currently not approved for use in pediatric patients and only a handful of prospective trials have been conducted. In 2007, Young et al. published a pilot dose-finding and safety trial in patients < 6 months of age with thrombosis (36). This study (*n* = 16) established pediatric dosing for bivalirudin of a bolus dose (0.125 mg/kg) followed by continuous infusion (starting at a rate of 0.125 mg/kg/h) to target 1.5–2.5 times the patient’s baseline aPTT. Two patients suffered from a major bleeding event. No patient had thrombus progression at 48–72 h and 6 patients (37.5%) had complete or partial resolution of the thrombus at 72 h. This was followed by the Utilization of Bivalirudin on Clots in Kids (UNBLOCK) study; an open-label, single-arm, dose-finding, pharmacokinetic, safety, and efficacy study conducted in children aged 6 months to 18 years with a deep venous thrombosis (37). Eighteen children received a bivalirudin bolus (0.125 mg/kg) followed by continuous infusion (starting at a rate of 0.125 mg/kg/h) to target 1.5–2.5 times the patient’s baseline aPTT. There were no major bleeding events, only one minor bleeding event and the only non-bleeding adverse event was hypertension. An interesting finding was the complete or partial thrombus resolution rate of 50% at 48–72 h and 89% at 25–35 days. While this finding supported a possible therapeutic benefit, the small number of children enrolled and lack of comparable data for UFH make it difficult to draw conclusions about efficacy benefits. In this study, the plasma bivalirudin levels correlated more closely with the infusion rate than with the aPTT, therefore aberrant aPTT results should be interpreted within the clinical context. A more detailed discussion regarding drug monitoring is found above. An additional prospective trial enrolled children who underwent PCI for congenital heart disease (*n* = 110) (38). In this trial, patients received a weight-based dose of 0.75 mg/kg bolus followed by a 1.75 mg/kg/h continuous infusion. In this setting, pharmacodynamics and kinetics were similar to adults with a trend toward increased clearance rates in neonates. There were minimal major bleeding events (1.8%) or thrombotic events (8.3%).

There is only 1 randomized trial of bivalirudin use in children to our knowledge. In this trial, bivalirudin was compared to UFH in children with acyanotic aged 1–12 years who underwent open-heart surgery (39) (*n* = 50). Bivalirudin dosing in this study was extrapolated from approved weight-based dosing in adults. Children receiving UFH achieved higher ACT levels at first bolus and 30 min after the onset of CPB (673 s vs. 458 s; *p* < 0.001 and 839 s vs. 590 s; *p* = 0.03) and a shorter duration of post-CPB ACT increment (immediately after CPB vs. 2 h; *p* < 0.01). Bivalirudin also prolonged the duration of surgery mostly due to the need for additional bolus doses each of which prolonged the surgery by 10–13 min. There was no difference, however, in chest tube output or need for transfusions between the two groups.

### Use in Mechanical Support Devices

Robust randomized or prospective data for the use of bivalirudin in mechanical support devices, such as ECMO circuits, and ventricular assist devices (VADs) are lacking. A systemic review from 2005 to 2017 looking at bivalirudin and ECMO found only 8 relevant publications (58 patients, 24 pediatric); 2 retrospective case-control studies, 1 case series, and 5 case reports; highlighting the knowledge gap in this area (40). In the two studies comparing bivalirudin to UFH, there was no difference in complication rates (41, 42), however, one study did show some advantages with lower blood loss and transfusion rates in the bivalirudin group (42). The variability across studies in ECMO likely reflects differences in the circuit, dosing of bivalirudin, limitations of retrospective data collection, and the heterogeneous population of patients placed on ECMO.

A small number of studies have reported on the clinical outcomes of in-circuit thrombosis rates, need for circuit exchange, and need for blood product replacement. In a retrospective chart review (*n* = 295), Rivosecchi et al. showed a decrease in circuit-related thrombotic events (32.7% vs. 17.3%; *p* = 0.003) with the use of bivalirudin in patients placed on veno-venous (VV)-ECMO (43). These results were similar to a prospective cohort study (20 ECMO runs in 18 patients) that showed that circuit interventions were lower in patients who received bivalirudin as compared to UFH [median (interquartile range; IQR) circuit intervention rate per run was (0–1) and (1–2); *p* = 0.0126] (43). It is important to note, however, that in this study, the comparison is within patients who received both UFH and bivalirudin, with 80% of patients were placed on bivalirudin only after UFH failure. A second retrospective study (*n* = 429) failed, however, to demonstrate a significant difference in the composite outcome of circuit intervention rate and oxygenator/pump change-out rate (44). One additional retrospective review compared adults who received UFH or bivalirudin treated per high- or low-intensity protocols (*n* = 72) (45). The authors found no difference with regards to thrombotic events during the initial 96 h, the course of the ECMO run, or at any time during the admission. When high-intensity UFH and bivalirudin dosing protocols were specifically compared, patients who received high-intensity bivalirudin were more likely to spend time in the therapeutic range than those being treated with high-intensity heparin, possibly related to the enhanced pharmacokinetics of bivalirudin or its lack of dependence on antithrombin. This finding did not translate into meaningful differences in clinical outcomes related to hemostasis and thrombosis. One pediatric retrospective study (*n* = 32) found no difference in time within the therapeutic range between UFH and bivalirudin (46). In this study, UFH resulted in higher amounts of iatrogenic blood loss per hour; however, this did not translate into higher product utilization. Lastly, no difference was seen in circuit changes between the two groups.

There is potential that the short half-life of bivalirudin, while desirable, may not be ideal for mechanical support devices where areas of stasis or non-systemic blood flow may result. This may result in disproportionately low bivalirudin concentrations and thrombus formation. With ECMO, there is no contractile force on blood flow allowing for cardiac blood stagnation and possible formation of intracardiac thrombus, especially in the setting of a very large right or left atrium with insufficient venous drainage or with very poor ventricular systolic function (47).

## Alternative Direct Thrombin Inhibitors

Bivalirudin is just one of the DTIs and it has the most expansive label indication. Intravenous DTIs represent a class of medications that can be either synthetic hirudin fragments (i.e., lepirudin and bivalirudin) or low-molecular-weight inhibitors that interact at the active site of thrombosis (i.e., argatroban). To our knowledge at this time, only a few case reports have described the use of lepirudin with the primary indication being HITT in the majority of the cases (48, 49). Lepirudin is not available in the US.

One potential benefit of argatroban over bivalirudin is the long half-life (45 min vs. 25 min) overcoming the potential limitation of bivalirudin in areas of stagnation addressed above. Agatroban undergoes liver metabolism and dosing is not renal dependent. Argatroban has successfully been used in the setting of ECMO. In propensity score-matched observational study of 78 adult patients who received UFH were matched to 39 patients who received argatroban. A composite primary outcome of major thrombosis and/or major bleeding was seen in 83% of patients with UFH and 79% of the patients who received argatroban. The authors concluded that argatroban was found to be non-inferior to UFH regarding bleeding and thrombosis rates. While argatroban drug costs were higher, they were balanced when accounting for blood product support and HIT testing associated with UFH use (50). A systematic review (*n* = 13) reporting on argatroban use in 307 patients with ECMO found considerable variation in dosing practice and target anticoagulation goals with either the aPTT or ACT. These differences are likely related to patient differences with regards to disease severity, end-organ function, and institutional aPTT or ACT goals. Across the included studies, bleeding and thromboembolic event rates were similar to UFH (51).

## Conclusion

Prevention of bleeding and thrombosis in the setting of the inherent variability in ECMO circuits, cannulation, and patient populations is extremely challenging. The choice of anticoagulants, which was limited to heparin, has now increased with the new parenteral anticoagulants. Bivalirudin is being increasingly explored for anticoagulation in patients with ECMO for its obvious advantages of short half-life and ability to bind to both free and clot-bound thrombin, but the lack of a reversal agent is a primary disadvantage. Although data are limited, there appears to be increasing evidence that this may at least be an equally efficacious. It also has the potential to avoid the use of antithrombin replacement and reduce lab monitoring. Current potential benefits are mostly extrapolated from adult data in the setting of PCI and additional studies are needed for specific ECMO to determine the true impact on clinical outcomes, such as transfusion needs, circuit-related thrombosis, and hemolysis. Randomized controlled trials are extremely difficult to conduct in this diverse population of patients and continued data collection on the safety and efficacy of the use of bivalirudin in ECMO will be required to determine if this can be considered a first-line anticoagulant in ECMO.

## Author Contributions

CN, MC, and CO reviewed the literature, wrote, edited, and revised the manuscript. All authors agreed with the final version of the manuscript.

## Conflict of Interest

The authors declare that the research was conducted in the absence of any commercial or financial relationships that could be construed as a potential conflict of interest.

## Publisher’s Note

All claims expressed in this article are solely those of the authors and do not necessarily represent those of their affiliated organizations, or those of the publisher, the editors and the reviewers. Any product that may be evaluated in this article, or claim that may be made by its manufacturer, is not guaranteed or endorsed by the publisher.
